# Supernumerary Formation of Olfactory Glomeruli Induced by Chronic Odorant Exposure: A Constructivist Expression of Neural Plasticity

**DOI:** 10.1371/journal.pone.0035358

**Published:** 2012-04-12

**Authors:** Pablo Valle-Leija, Eduardo Blanco-Hernández, Rene Drucker-Colín, Gabriel Gutiérrez-Ospina, Roman Vidaltamayo

**Affiliations:** 1 Departamento de Neuropatología Molecular, Instituto de Fisiología Celular, Universidad Nacional Autónoma de México, México Distrito Federal, México; 2 Departamento de Biología Celular y Fisiología, Instituto de Investigaciones Biomédicas y Coordinación de Psicofisiología, Facultad de Psicología, Universidad Nacional Autónoma de México, México Distrito Federal, México; 3 Departmento de Ciencias Básicas, Centro de Diagnóstico Molecular y Medicina Personalizada, Universidad de Monterrey, Garza García, Nuevo León, México; Duke University, United States of America

## Abstract

It is accepted that sensory experience instructs the remodelling of neuronal circuits during postnatal development, after their specification has occurred. The story is less clear with regard to the role of experience during the initial formation of neuronal circuits, whether prenatal or postnatal, since this process is now supposed to be primarily influenced by genetic determinants and spontaneous neuronal firing. Here we evaluated this last issue by examining the effect that postnatal chronic exposure to cognate odorants has on the formation of I7 and M72 glomeruli, iterated olfactory circuits that are formed before and after birth, respectively. We took advantage of double knock-in mice whose I7 and M72 primary afferents express green fluorescent protein and β-galactosidase, correspondingly. Our results revealed that postnatal odorant chronic exposure led to the formation of permanent supernumerary I7 and M72 glomeruli in a dose and time dependent manner. Glomeruli in exposed mice were formed within the same regions of olfactory bulb and occupy small space volumes compared to the corresponding single circuits in non-exposed mice. We suggest that local reorganization of the primary afferents could participate in the process of formation of supernumerary glomeruli. Overall, our results support that sensory experience indeed instructs the permanent formation of specific glomeruli in the mouse olfactory bulb by means of constructivist processes.

## Introduction

Unraveling the mechanisms by which neuronal circuits in sensory systems are formed and refined until reaching their mature configuration has been one of the major challenges in the field of developmental neurobiology. Based on the seminal work conducted in the cat and monkey's visual system by Hubel and Wiesel during the 1960's and 70's and on more recent developments, most contemporary neurobiologists would agree that sensory experience plays an instructional role on sensory circuit refinement and plasticity [1]–[11]. The story is not different in the rodent olfactory bulb where the experimental data available support the notion that the organization and refinement of the anatomical layout of these circuits are highly sensitive to experience [12]–[16]. Nevertheless, the participation of experience during the initial formation of sensory circuits is still under debate because the evidence so far published supports that it is the spontaneous activity going on across related neuronal assemblies what instructs the early specification and formation of such circuits during the ontogeny [5], [17]–[20]. Accordingly, in the olfactory bulb, the suppression of spontaneous firing in a selective subset of olfactory sensory neurons prevents the formation of their corresponding circuits [18]. In spite of these results, evidence showing mice displaying odorant preference and enlarged glomerular size following the prenatal and early postnatal exposure to acetophenone and isopropyl tiglate [21], opens the possibility that sensory experience may indeed influence brain circuit formation earlier than previously thought. In this regard, the olfactory bulb seems an adequate model to explore influences of early sensory experience on circuit specification and formation since some olfactory glomeruli are formed before birth, but some others form postnatally [22]. The fact that primary afferents associated with specific olfactory sensory neurons in the olfactory mucosa converge onto specific, easily identifiable glomeruli [13], [23], [24], provides with a unique opportunity to assess the effects of sensory experience on the initial steps of glomeruli formation using exposure to distinct odorants. Hence, in this paper we took advantage of both features to monitor the formation of specific glomeruli following chronic odorant exposure using mice having I7 and M72 olfactory receptors (ORs) tagged with green fluorescent protein (I7tauGFP) and β-galactosidase (M72tauLacZ), respectively. Specific stimulation was achieved by exposing the mice to heptaldehyde or acetophenone, the cognate ligands of I7 and M72 ORs, respectively [24]–[26].

## Results

### Chronic exposure to cognate odorants during the first twenty days of life induced the formation of odor-specific supernumerary glomeruli

**Figure 1:**
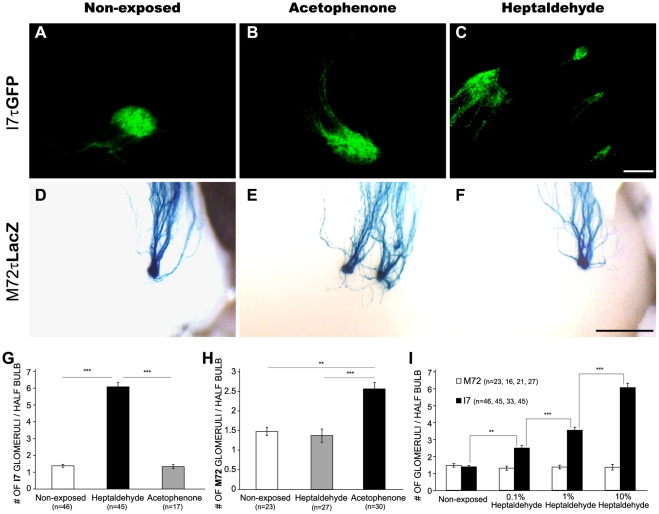
Chronic odorant exposure led to the dose-dependent and circuit-specific formation of supernumerary glomeruli. Figures A–C show representative I7tauGFP glomeruli from non-exposed mice and from those exposed to pure acetophenone and 10% heptaldehyde, the latter being the cognate ligand. Figures D–F show representative M72tauLacZ glomeruli from non-exposed mice and from those exposed to pure acetophenone and 10% heptaldehyde, the former being the cognate ligand. Scale bar 100 µm (I7tauGFP) and 500 µm (M72tauLacZ). The bar graphs in G and H show the average number of I7 and M72 glomeruli per half bulb, respectively, for each condition. The bar graph in I depicts the average number of I7 and M72 glomeruli in response to different concentrations of heptaldehyde. Kruskal-Wallis sum rank test: p<0.01 followed by Dunn multiple comparison test: **, P<0.01; ***, P<0.001.

**Figure 2:**
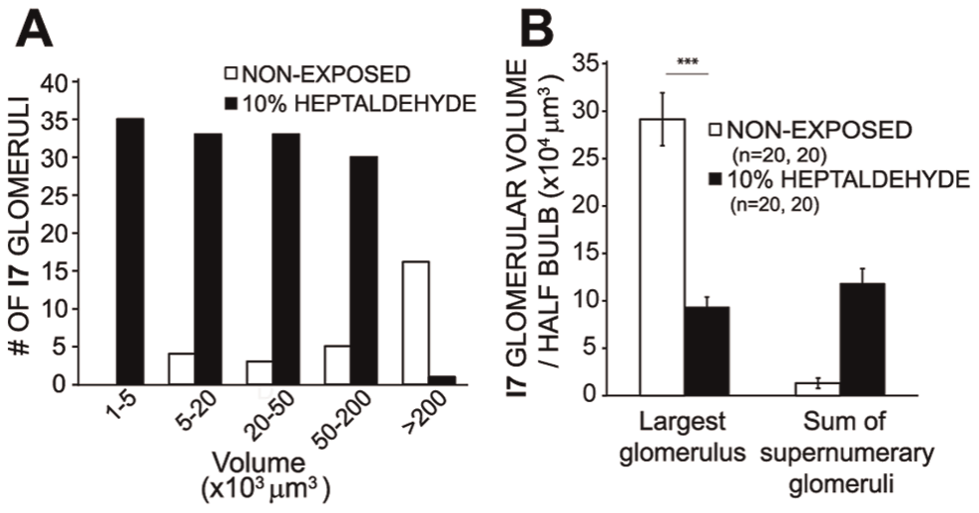
Chronic odorant exposure led to the redistribution of the glomerular volume. The graph in A shows a volume histogram of the total population of I7 glomeruli in 20 half bulbs of non-exposed mice and in those exposed to 10% heptaldehyde. The bar graph in B depicts the average volume of the largest I7 glomerulus per half bulb and the average sum of the volume of I7 supernumerary glomeruli per half bulb in non-exposed mice and in those exposed to 10% heptaldehyde. Kruskal-Wallis sum rank test: p<0.01 followed by Dunn multiple comparison test: ***, P<0.001.

The effect of chronic odorant exposure on olfactory glomerular formation was first evaluated by continuously exposing I7tauGFP/M72tauLacZ mice to either heptaldehyde or acetophenone, from birth to 20 days of age. In both cases, chronic exposure to the cognate odorant bilaterally increased the number of specific glomeruli in the olfactory bulb (Figure 1). This effect was odor-specific since neither heptaldehyde nor acetophenone increased the number of M72 or I7 glomeruli, respectively (Figure 1). Also, the fact that all the results were observed in double knock-in mice having the same genetic background and the formation of supernumerary glomeruli was dose-dependent further support the specificity of the response (Figure 1I). An interesting finding was that most glomeruli in exposed mice were small in size (Figure 2A and 2B), one half of their total volume in each half bulb was confined to one glomerulus (named Largest) approximately, and the other half corresponded to supernumerary glomeruli (Figure 2B). Equally important was that supernumerary glomeruli always occupied locations closed (<700 µm linear distance) to the largest glomerulus, thus emphasizing their relatedness. Interestingly, most bulbs analyzed showed I7 glomeruli already formed at birth (Figure S1). This suggests that supernumerary glomeruli can be formed after the initial formation of the I7 glomerulus, as supported by the observation made in mice exposed after postnatal day (PD) 5 and PD10 (Figure S2).

### Supernumerary glomeruli persisted into adulthood and continued to grow after odorant exposure

**Figure 3:**
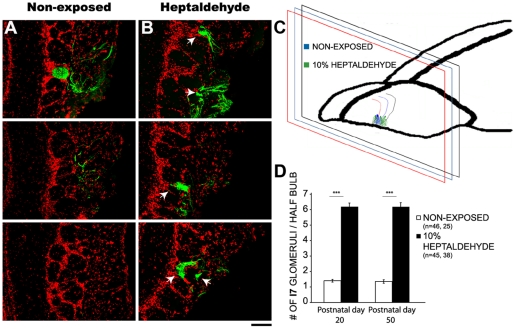
Supernumerary glomeruli persisted after interrupting chronic odorant exposure. Figures A and B show representative I7tauGFP glomeruli (green) in coronal brain slices stained with DAPI (red) from non-exposed mice and from those exposed to 10% heptaldehyde and later raised without the odorant until postnatal day 50. Arrowheads indicate supernumerary glomeruli. Scale bar 100 µm. Figure in C shows a schematic side view representation of the olfactory bulb, indicating the anterior ventrolateral position of I7 glomeruli in the coronal plane in non-exposed mice and in those exposed to 10% heptaldehyde, according to the location across brain slices. Bar graph in D shows the average number of I7 glomeruli per half bulb 30 days after the end of exposure. Kruskal-Wallis sum rank test: p<0.01 followed by Dunn multiple comparison test: ***, P<0.001.

**Figure 4:**
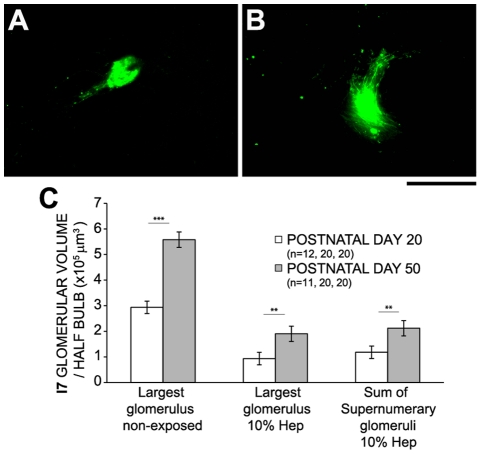
Supernumerary glomeruli grew in size after interrupting chronic odorant exposure. Figures A and B show representative I7tauGFP supernumerary glomeruli at postnatal day (PD) 20 and at PD50, respectively, from 10% heptaldehyde exposed mice. Scale bar 100 µm. Bar graph in C depicts the average I7 glomerular volume per half bulb, at PD20 and PD50, of the largest glomerulus in non-exposed mice, and the largest glomerulus and the sum of supernumerary glomeruli in mice exposed to 10% heptaldehyde (10% Hep). Non paired t-test: **, P<0.01; ***, P<0.001.

Glomerular elimination has been claimed to be a common feature during olfactory bulb postnatal remodelling [12], [13], although see [27]. Hence, we examined whether I7 and M72 supernumerary glomeruli induced by chronic exposure to cognate odorants endure as animals mature, even if I7tauGFP/M72tauLacZ mice were no longer exposed to the corresponding odorants. In both cases, the number of supernumerary glomeruli not only remained constant (Figure 3 shows data for I7 glomeruli, not for M72 glomeruli), but underwent significant growth in size (Figure 4). Similarly, non-exposed mice kept constant the number of glomeruli throughout their life (Figure S3). Additionally, in adulthood, the spatial position of glomeruli in exposed mice was approximately the same as non-exposed mice (Figure 3C).

### The specific formation of supernumerary glomeruli occurred during a sensitive period of postnatal development

**Figure 5:**
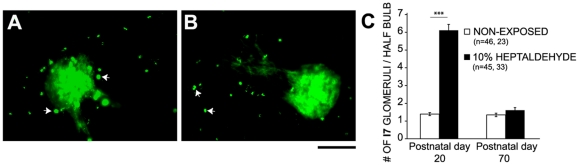
Adult mice were unable to form supernumerary glomeruli in response to chronic odorant exposure. Figures A and B show representative I7tauGFP glomeruli from non-exposed mice and from those exposed to 10% heptaldehyde, respectively; periglomerular neuron autofluorescence (arrows) is commonly seen in adults. Scale bar 100 µm. The bar graph in C depicts the average number of I7 glomeruli per half bulb in mice exposed during early postnatal stage (PD0–PD20) and in those exposed during adulthood (PD50–PD70). Mann-Whitney U test: ***, P<0.001.

Sensory experience often modulates the development of sensory projections within a restricted temporal window [1], [5], [12], [13]. We then compared the likelihood to induce the formation of supernumerary I7 glomeruli in the adult mice. Adults exposed to the highest heptaldehyde concentration during 20 days were unable to increase the number of I7 glomeruli by the end of exposure period (Figure 5).

### Local primary afferent reorganization occurred during chronic odorant exposure

**Figure 6:**
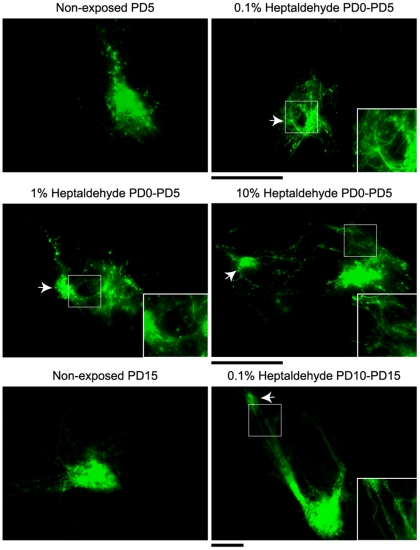
The largest glomeruli displayed primary afferent reorganization within a restricted space during chronic odorant exposure. The figure shows the expression of I7tauGFP glomeruli in non-exposed mice and mice exposed to heptaldehyde. Notice that primary afferents redistribute in variable ways between the largest and supernumerary glomeruli (arrows) in exposed mice. The effect was seen in all half bulbs analyzed (n = 10 for each condition) when mice were exposed from postnatal day (PD) 0 to PD5. In mice exposed from PD10 to PD15 the effect was seen in 8 out of 10 total half bulbs analyzed. Scale bar 100 µm; inset square is 2× magnification.

Volume redistribution and topological closeness among supernumerary I7 and M72 glomeruli suggested that redirected, but precise, local primary afferent growth could underlie the formation of supernumerary I7 and M72 glomeruli in odorant exposed mice. Accordingly, primary afferents associated to the I7 circuit showed a single target spot in the olfactory bulb of non-exposed mice at postnatal day (PD) 5 (Figure 6). In contrast, heptaldehyde exposed mice displayed afferents coming out from the largest glomerulus that targeted what seemed to be secondary glomeruli. This was true for mice exposed to different doses of heptaldehyde between PD0 and PD5 (Figure 6). The same phenomenon was also observed in mice exposed between PD10 and PD15. These observations then support that axonal reorganization could contribute to supernumerary glomeruli formation and that olfactory primary afferents displayed a protracted phase of axonal growth that lasted for at least two weeks after birth under the experimental conditions tested (Figure 6). Another related finding was that even though, in exposed mice, the largest glomerulus and supernumerary glomeruli increased their volume as postnatal development proceeded (Figure 4C), the total glomerular volume was not greater than the volume of the corresponding glomerulus in non-exposed mice, at least by PD20 (Figure S4). This could mean that primary afferents re-distribute the volume between the largest and supernumerary glomeruli.

## Discussion

It is well established that sensory experience instructs the refinement and plasticity of sensory circuits in the developing brain [1]–[11]. The initial formation and overall layout of neuronal circuits, however, is thought to heavily depend upon spontaneous patterns of neuronal activation across the different synaptic relays that constitute each developing sensory pathway [5], [17]–[20], including the olfactory system [18]. In spite of these ideas, the present work provides evidence supporting that early olfactory experience instructs the formation of olfactory glomeruli in a way that is odor and circuit specific. Indeed, heptaldehyde and acetophenone exposure during the first twenty days of life led to the formation of permanent supernumerary I7 and M72 olfactory glomeruli in mice, respectively. Exposure to acetophenone did not result in supernumerary I7 glomeruli nor did supernumerary M72 glomeruli to heptaldehyde. These adjustments took place only when mice were exposed when young, and seemed to depend on the salience of the olfactory stimuli since their occurrence was dose dependent. In addition, not only these supernumerary glomeruli endured over time, but they also grew significantly as animals mature. The fact that I7 and M72 glomeruli are formed pre- (Figure S1) and postnatally [22], [28], respectively, suggests that chronic olfactory experience before, during or after glomerular formation (Figure 1, 6 and S2) promotes the formation of a greater number of olfactory iterated circuits presumably destined to the processing of information associated with salient specific odors.

Our study, nonetheless, is not the first in reporting increased number of specific glomeruli in mice. They frequently occur in mice that have been genetically engineered to alter the expression of axon guidance molecules [29]. Hence, our results may in part be explained by differences in axon guidance molecules availability, repulsion and/or adhesion signals [30]. In support of this possibility, we documented volume redistribution and topological closeness among supernumerary I7 and M72 glomeruli, as well as the local primary afferent reorganization in mice exposed chronically to cognate odorants. Complementarily or alternatively, our results can also be attributed to modified patterns of spontaneous and/or increased or reduced presynaptic and/or postsynaptic neuronal activity [12], [14], [18], [31]–[34], shifts in the availability of cAMP associated with OR activation [34]–[36], differential activation of G protein and/or adenylate cyclase [35]–[37] and/or changes in BDNF availability [14], [38]. All the named factors are known to greatly influence the degree of glomerular axon coalescence and arborization, and glomeruli spatial distribution and number. The merit of these possibilities deserve consideration in future studies.

Unlike other similar studies that analyzed the effect of odorant exposure through the mother, during fetal and early postnatal stage [13], [21], our study deals with the effect of mother independent odorant exposure or non-conditioned exposure [13]. The importance of this is in relation to the differing effects in glomerular number and size. Odorant exposure through the mother causes an increase in size of the activated glomeruli and accelerates their refinement [13], [21], while our results show a decrease in size and increase in number of the nominally activated glomeruli in response to chronic postnatal odorant exposure (see [39] for similar results for P2 glomeruli). The reasons for such a discrepancy are unclear. However, laboratory-to-laboratory differences in the number of P2 and M72 glomeruli in the olfactory bulb are commonly reported [12], [37], [40]–[44], a circumstance that has been suggested to be due to differences in the olfactory environment [39]. Thus, future studies must compare different protocols of odor exposure to evaluate differential plastic responses across the population of glomeruli, among other possibilities, as suggested by [21].

Traditionally, developmental neurobiologists have favored the selectionist view of the formation of neuronal circuits during brain development. There is evidence, however, supporting that many regions of the mammalian brain built up their circuits by adding new connectivity with little evidence of selection [17], [45]. The olfactory bulb is not excluded from this debate. Although previous work has suggested that neuropil complexity within olfactory gomeruli increases and the number of glomeruli continuously augments as postnatal development proceeds [27], other calls pintpoint otherwise suggesting that olfactory glomeruli number decrease through fiber elimination or remodelling at a local level [12], [13], [32]. The present results indicate that during plastic responses to chronic odorant exposure, supernumerary glomeruli are added and consolidated as the olfactory bulb matures; no glomerular elimination was seen under any experimental condition (Figure 3 and S3). This result is compatible with the idea that at least under chronic odorant exposure before, during or after glomerular formation, the olfactory bulb uses a constructivist strategy, both at the circuit and glomerular levels, to respond to increased sensory demands; however, this process did not involve an increase of total glomerular volume (Figure 2 and S4); also see [14], [39]. In summary, the present results document a new strategy for the developing olfactory bulb to engange plastic responses.

## Materials and Methods

### Animals

I7-IRES-tau-GFP and M72-IRES-tau-LacZ knock-in mouse strains were generously gifted by Dr. Peter Mombaerts. Crossbreeding of both lines was made to obtain a double marked I7tauGFP/M72tauLacZ mouse strain in which all experiments were done. The expression of the axonal marker tau-green fluorescent protein and tau-beta-galactosidase are under the direct control of the endogenous OR promoter, for more details about the mice see [24], [42]. Mice were mantained in a temperature controlled vivarium on a 12/12 light-dark cycle with food and water available *ad libitum*. For the experiments during adulthood, mice were weaned at 21 days of life. Animal procedures followed the National Institutes of Health (NIH) guidelines for the care and use of experimental animals (NIH approval number A5281–01). The protocols were revised and approved by the local animal rights committee at the Universidad Nacional Autónoma de México. Each experimental group came from at least two mouse litters and experiments were analyzed blindly by a single observer. All efforts were made to minimize the number of animals used and their potential suffering.

### Chronic odorant exposure

I7tauGFP/M72tauLacZ mice were exposed to either heptaldehyde (95%; Sigma-Aldrich) or acetophenone (98%; J.T. Baker). Heptaldehyde and acetophenone are the cognate ligands of I7 and M72 ORs, respectively [24]–[26]. Pregnant female mice were housed in closed-top cages (1/cage), after pups were born, odorant stimulation was achieved at different postnatal stages. Each cage had a tight-sealed bottle from which the odorant was released via a filter paper; the bottle was inaccessible to mice. The odorant solution was changed daily to maintain proper concentration. To make odorant availability as equivalent as possible, we used heptaldehyde concentrated ten times lower than acetophenone because its vapor pressure is approximately ten times higher (Heptaldehyde: 3.85 mm/Hg; Acetophenone: 0.44 mm/Hg at 25°C). For acetophenone exposure, odorant was supplied from postnatal day (PD) 0, just after birth, to PD20 (98% concentrated). For heptaldehyde exposure, odorant was supplied to different animal groups from PD0 to PD20 (0.1, 1 and 10% concentrated), PD0 to PD5 (0.1, 1 and 10% concentrated), PD10 to PD15 (0.1% concentrated) and PD50 to PD70 (10% concentrated). Half of the mice that were exposed to 10% heptaldehyde from PD0 to PD20 were inmediatly sacrificed, and the other half were raised without the odorant until PD50, in different cages to prevent odorant contamination. In all of the experiments with heptaldehyde, pure DMSO (J.T. Baker) was used for diluting it. Mice from acetophenone, heptaldehyde and non-exposed groups were raised in different rooms in a vivarium with closed circuit ventilation system to prevent cross effects.

### Imaging of I7tauGFP glomeruli in fresh tissue

Mice were sacrificed by decapitation and olfactory bulbs were dissected. Then, the bulbs were mounted onto glass slides, coverslipped and flattened (approximately 110 µm thick) to improve GFP visualization. Olfactory I7tauGFP glomeruli were identified by using epiﬂuorescence and visualized through a Leica DM6000 vertical microscope. Images were captured (20X and 40X) and digitized with the software Leica LAS AF. The olfactory bulbs were imaged as early as 10 minutes and as late as 2 hours after sacrifice without apparent degeneration of the structures or decrease of the intensity of GFP fluorescence. Only glomeruli with undamaged axons were considered for analyses. The morphological parameters used to consider the observed structures as I7tauGFP glomeruli were them having a volume of at least 1000 µm^3^ and intact fibers coalescing onto them. The search and counting of I7tauGFP glomeruli and the distance between them was done online in the microscope across all the visual field, structures smaller than 1000 µm^3^ were excluded from the analysis offline after image capturing. Volume values were estimated offline in stacks (1 µm thick) using the ImageJ (NIH) Volumest plugin [46].

### Imaging of I7tauGFP glomeruli with immunofluorescence

Mice were deeply anesthetized with sodium pentobarbital and perfused transcardially with PBS followed by freshly prepared 4% paraformaldehyde. After post-fixation for 3 hours, brains were cryoprotected in buffered sucrose (30%) for 2 days at 4°C. Brain slices (20 µm) were obtained with a cryostate. Sections were rinsed in 0.1 M PBS and incubated with a blocking solution containing 10% of normal horse serum and 0.3% Triton X−100 for 1 hour. To label I7tauGFP positive structures, sections were incubated with a polyclonal goat anti-GFP antibody (Abcam, Cat. No. ab6673) at 1∶1,000 dilution in blocking solution for 24 hours at 4°C. Then, sections were rinsed and incubated for 2 hours with a donkey anti goat-Alexa 555 secondary antibody (Invitrogen) and counterstained with DAPI. Slides were then coverslipped using DACO. Immunofluorescence was achieved for non-exposed mice at PD50 and mice exposed to 10% heptaldehyde during PD0 to PD20 and raised until PD50. I7tauGFP immunofluorescent structures were visualized through a Leica DM6000 vertical microscope. Images were captured (20X) and digitized with the software Leica LAS AF, and used only for figure representation.

### Imaging of M72tauLacZ glomeruli with X-gal staining

M72tauLacZ glomeruli were revealed in whole mounts [40]. Mice were deeply anesthetized with sodium pentobarbital and perfused transcardially with PBS followed by freshly prepared 4% paraformaldehyde. Whole mounts were prepared and the medial or the dorsal aspect of the olfactory bulb was imaged. To reveal β-galactosidase activity, the samples were incubated for 5 hours in a phosphate buffer containing 2 mM MgCl_2_, 0.01% sodium desoxycholate, 0.02% Nonidet P40, 5 mM potassium ferricyanide, 5 mM potassium ferrocyanide and 1 mg/ml of X-gal. Then the samples were washed thoroughly for 10 min in PBS and images of M72tauLacZ glomeruli were captured through a stereoscopic microscope Leica EZ4D. The parameter used to consider the observed structures as M72tauLacZ glomeruli was them having at least 5 visible fibers or one or more fiber bundles reaching them. The counting was done offline after image capture and digitization with Leica FireCam software.

### Statistical analysis

Statistics were performed in GraphPad Prism 5. Quantitative data are presented as averages ±standard errors. The statistical analysis was done by taking into account every half bulb independently in all mice [12], [13], because the probability of finding “n” number of glomeruli per half bulb variated equally between the half bulbs of the same mouse than between different mice. Analyses were performed blindly by a single observer.

## Supporting Information

Figure S1 **Mice at birth have 64% of their half bulbs with I7 glomeruli formed.** Figures A and B show I7tauGFP positive axons and glomerulus, respectively, just after birth. Scale bar 100 µm. We observed two different possibilities: half bulbs with locally dispersed I7tauGFP axons but with no coalescence (A), and half bulbs with a high degree of coalescence of I7tauGFP axons, forming glomeruli (B). We qualitatively separated both populations, and found that 22 of 34 half bulbs (64%) had one I7tauGFP protoglomerulus formed (C).
(TIF)

Figure S2 **Chronic odorant exposure after postnatal day (PD) 5 and PD10 led to the formation of supernumerary glomeruli.** Figures A and B show I7tauGFP supernumerary glomeruli after exposure to 10% heptaldehyde (10% Hep). Scale bar 100 µm. The bar graphs in C show the average number of I7 glomeruli per half bulb for each experimental condition. Kruskal-Wallis sum rank test: p<0.01 followed by Dunn multiple comparison test: ***, P<0.001.
(TIF)

Figure S3 **No refinement of I7 and M72 glomeruli was seen across postnatal development in a control olfactory environment.** The average number of I7 and M72 glomeruli per half bulb remained constant during early postnatal stage and adulthood in non-exposed mice. Kruskal-Wallis sum rank test: p>0.05.
(TIF)

Figure S4 **Chronic odorant exposure did not led to increase of total glomerular volume.** The bar graph shows total I7 glomerular volume per half bulb for non-exposed mice and mice exposed to heptaldehyde from postnatal day (PD) 0 to PD20. ANOVA: p<0.05 followed by Dunnett multiple comparison test: *, P<0.05.
(TIF)
