# Supplementary figures and images for: Supernumerary Formation of Olfactory Glomeruli Induced by Chronic Odorant Exposure: A Constructivist Expression of Neural Plasticity
Source: PLoS One. 2012 Apr 12;7(4):e35358. doi: 10.1371/journal.pone.0035358 (PMC3325210; doi:10.1371/journal.pone.0035358)

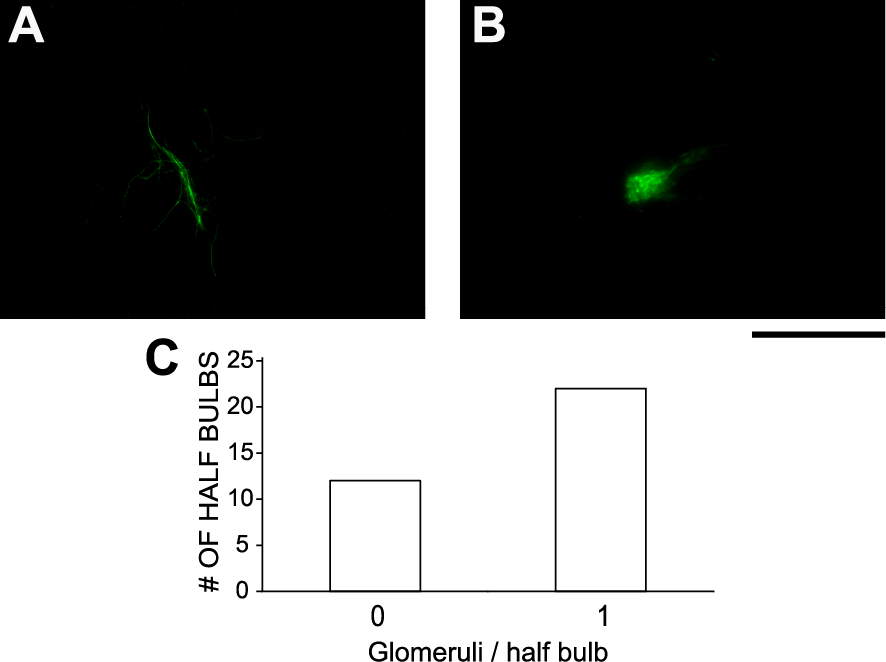

Supplement: Figure S1 — Mice at birth have 64% of their half bulbs with I7 glomeruli formed. Figures A and B show I7tauGFP positive axons and glomerulus, respectively, just after birth. Scale bar 100 µm. We observed two different possibilities: half bulbs with locally dispersed I7tauGFP axons but with no coalescence (A), and half bulbs with a high degree of coalescence of I7tauGFP axons, forming glomeruli (B). We qualitatively separated both populations, and found that 22 of 34 half bulbs (64%) had one I7tauGFP protoglomerulus formed (C). (TIF) [file pone.0035358.s001.tif]

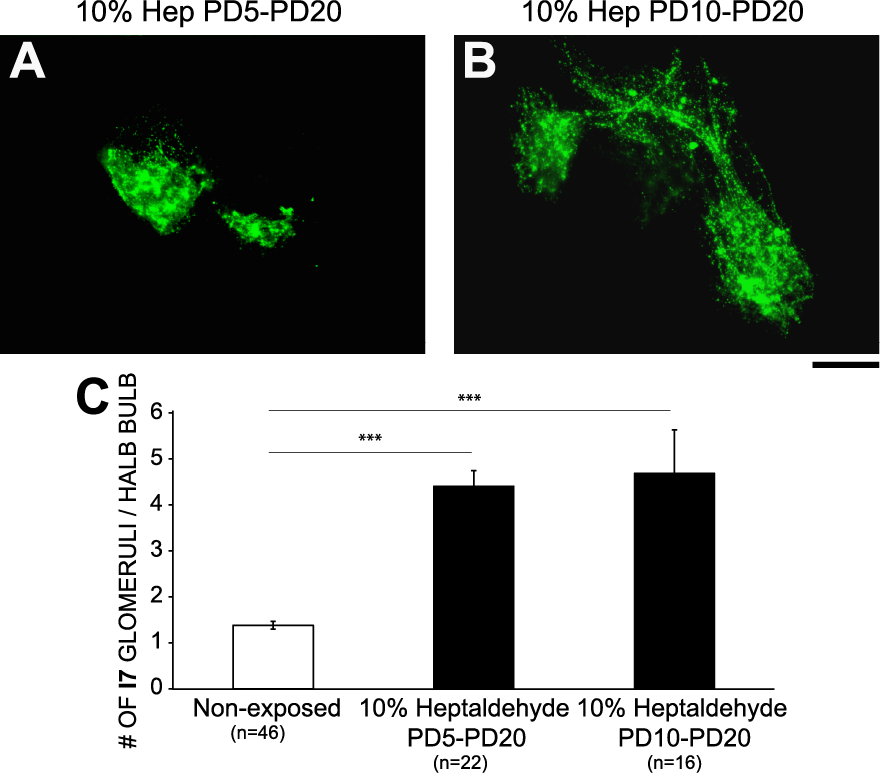

Supplement: Figure S2 — Chronic odorant exposure after postnatal day (PD) 5 and PD10 led to the formation of supernumerary glomeruli. Figures A and B show I7tauGFP supernumerary glomeruli after exposure to 10% heptaldehyde (10% Hep). Scale bar 100 µm. The bar graphs in C show the average number of I7 glomeruli per half bulb for each experimental condition. Kruskal-Wallis sum rank test: p<0.01 followed by Dunn multiple comparison test: ***, P<0.001. (TIF) [file pone.0035358.s002.tif]

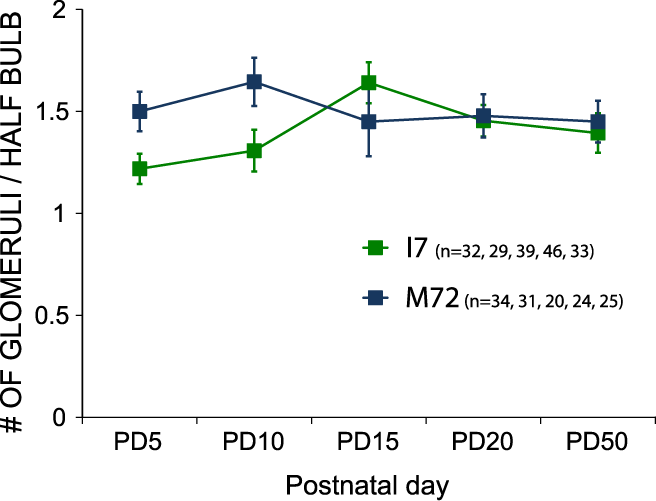

Supplement: Figure S3 — No refinement of I7 and M72 glomeruli was seen across postnatal development in a control olfactory environment. The average number of I7 and M72 glomeruli per half bulb remained constant during early postnatal stage and adulthood in non-exposed mice. Kruskal-Wallis sum rank test: p>0.05. (TIF) [file pone.0035358.s003.tif]

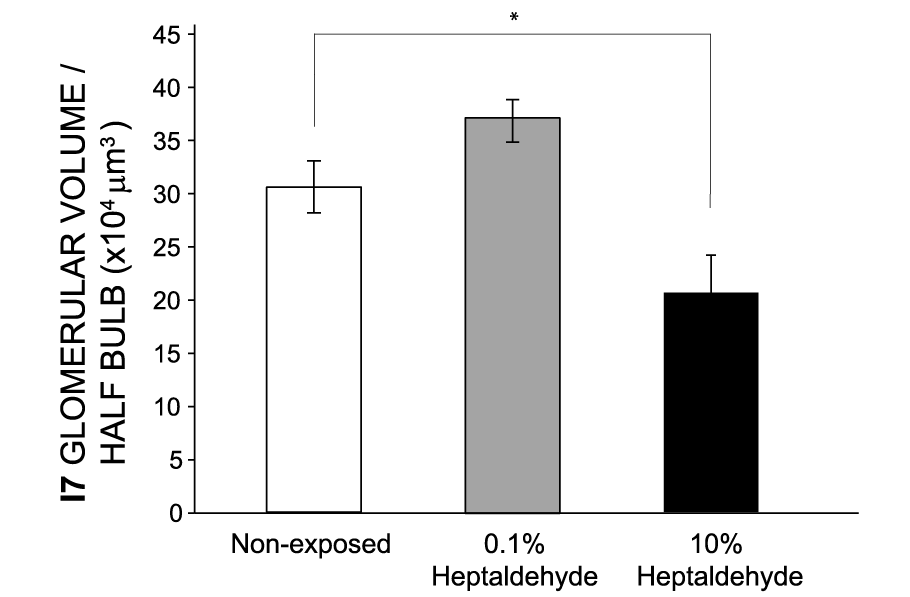

Supplement: Figure S4 — Chronic odorant exposure did not led to increase of total glomerular volume. The bar graph shows total I7 glomerular volume per half bulb for non-exposed mice and mice exposed to heptaldehyde from postnatal day (PD) 0 to PD20. ANOVA: p<0.05 followed by Dunnett multiple comparison test: *, P<0.05. (TIF) [file pone.0035358.s004.tif]
